# Design and numerical simulation for the development of an expandable paediatric heart valve

**DOI:** 10.1177/0391398820977509

**Published:** 2020-12-10

**Authors:** Monica M Kerr, Terence Gourlay

**Affiliations:** Department of Biomedical Engineering, University of Strathclyde, Glasgow, UK

**Keywords:** Aortic valves, artificial kidney, apheresis and detoxification techniques, modelling, cardiovascular, computer simulation, paediatric cardiac surgery, valves

## Abstract

Current paediatric valve replacement options cannot compensate for somatic growth, leading to an obstruction of flow as the child outgrows the prosthesis. This often necessitates an increase in revision surgeries, leading to legacy issues into adulthood. An expandable valve concept was modelled with an inverse relationship between annulus size and height, to retain the leaflet geometry without requiring additional intervention. Parametric design modelling was used to define certain valve parameter aspect ratios in relation to the base radius, *R*_b_, including commissural radius, *R*_c_, valve height, *H* and coaptation height, *x*. Fluid-structure simulations were subsequently carried out using the Immersed Boundary method to radially compress down the fully expanded aortic valve whilst subjecting it to diastolic and systolic loading cycles. Leaflet radial displacements were analysed to determine if valve performance is likely to be compromised following compression. Work is ongoing to optimise valvular parameter design for the paediatric patient cohort.

## Introduction

Congenital heart disease (CHD) affects eight in every 1000 live births. It is commonly complex in nature and often involves valvular lesions such as severe stenosis and atresia, contributing to cyanotic heart disease and poor prognosis if untreated. Valvular lesions can arise at birth due to complex congenital heart defects such as tetralogy of Fallot and hypoplastic left heart syndrome, at onset following trauma or disease such as rheumatic fever. Presently, native valve repair is the preferred treatment option but when this fails, patients require valvular replacement to mitigate symptoms associated with a stenotic, regurgitant or absent heart valve. Existing mechanical and bioprosthetic valves designed for the adult patient cohort cannot compensate for somatic growth, leading to complications associated with patient-prosthesis size mismatch including thrombo-embolism, stenosis, endocarditis and accelerated calcification.^1–4^ Growth of paediatric valvular prostheses is not a novel idea; the Ross procedure, is the most favourable treatment option in children as it facilitates aortic root growth, with actuarial patient survival rates of 86%–93% at 10 years.^5,6^ However, progressive root dilation of the pulmonary autograft is a commonly reported problem.^7,8^ More recently, tissue engineered heart valves have garnered a lot of excitement due to their potential to ‘grow with the child’. Yet real clinical success in this approach has been hampered by issues including incomplete decellurisation and tissue degeneration.^9^ Inevitability, these issues necessitate repeat surgeries throughout childhood, with each revision carrying with it an increased risk and associated mortality.^10^ Compounded by multiple revision surgeries needed to correct complex cardiac defects, and age-specific impaired quality of life, this can lead to far reaching legacy issues in adulthood.^11^

Emerging transcatheter valve technologies have developed an additional arm in paediatric valve replacement. The Melody transcatheter pulmonary valve has been used in pre-existing conduits to ease ventricular dilation in right ventricular outflow tract (RVOT) dysfunction. These valves are inserted surgically with the capacity to expand through intermittent balloon dilation during the somatic growth period. However, stent fracture of the Melody valve has been frequently observed, with freedom from fracture occurring in 77% and 60% of cases after 14 and 39 months respectively, caused mainly by severe RVOT obstruction or compression of the valve. Pre-stenting with a bare-metal stent or valve placement in an existing valve system was found to reduce the risk of TPV stent fracture although this procedure can lead to an increased conduit gradient.^12,13^ The Edwards SAPIEN transcatheter valve has also recently been deployed in RV to PA conduits although high migration rates have been reported due to the shorter stent size.^14^

We believe that the ideal paediatric heart valve is yet to exist and relies on the following design criteria:

1. A truly percutaneous heart valve, small enough to be introduced via paediatric iliac or femoral arteries. To achieve this, the leaflets of the valve must be thin enough (<100 µm) to fold down into a delivery device. Work is ongoing to develop an ultrathin transcatheter heart valve using carbon nanotube reinforced composites.^15^
2. Have a gradual annular expansion at a rate mimetic to somatic growth, mitigating the issues associated with PPM and accelerated calcification. This would eliminate the need for additional clinical intervention such as balloon dilation.
3. Expand radially without compromising coaptation and with minimal leaflet buckling which could cause excessive stresses and leakage in irregularly paired leaflets.

Several authors have reported on the relationship between various parameters of the native heart valve to ensure a fully competent valve.^16–18^ These parameters such as height, leaflet length and angle to the horizontal when closed, can be used as a baseline when developing a prosthetic heart valve that expands whilst respecting parameter ratios.^19^ Valve parameters described in previous studies on the native aortic valve are defined and their relationship to each other will be adapted to ensure a leaflet can be modelled that expands gradually across various diameters without compromising performance.

## Methods

### Valvular parametric design

The typical design of the transcatheter aortic valve (TAV) comprises of three semi-lunar shaped cusps that come together at their top edge to seal the valve. The leaflets are typically sutured to a compressible stent made of a shape memory alloy or stainless steel at their rounded edge. The proposed expandable paediatric valve is a polymeric composite TAV that does not stretch with size to match somatic growth. Rather, the valve will be designed at its maximal annular dimension and the leaflet free edge (*l*_f_) will remain taut during coaptation. Therefore, as the valve radius decreases, the angle (θ) of the free edge to the horizontal increases, resulting in a sagging leaflet belly. To achieve this, the relationships between valve parameters were coded in Fortran and investigated, using a similar approach to a geometrical modelling study by Labrosse et al.^19^ The variable parameters were valve height (*H*), coaptation height (*x*), and angle of stent to the vertical (*β*) so that they were exclusively dependent on the valve diameter and independent of the free edge length which is expected to remain constant and is defined as: $l_{f}=\frac{2\pi r_{\mathrm{cmax}}}{3}$

where, *r*_cmax_ is the radius of the commissures when at its maximum valve size (24 mm). The difference between the two radii, *r*_cmax_ and *r*_bmax_ (maximum base radius) produced a constant angle, β of 7.3°. This should, theoretically enable the leaflets to open beyond the boundary of the commissures and minimise the risk of buckling and obstruction to flow. Studies by Swanson and Clark^18^ and Thubrikar et al.^17^ determined an *R*_b_/*R*_c_ ratio of 1.2 and 1.0 respectively. Here, an *R*_b_/*R*_c_ ratio of 1.1 will be used.

Secondly, the maximum coaptation height (*x*) will be at its smallest when the valve is at its largest diameter and increase during radial compression. Typically, the coaptation area increases with size, and a parameter ratio of 0.17 in relation to diameter has previously been reported.^18^ However, as the same large leaflet surface area will be retained at smaller diameters, it is necessary for our TAV to have a coaptation area that increases with decreasing diameter. A ratio of 0.125 was therefore used at maximum diameter and predicted to increase to 0.25 at minimum diameter.

Thirdly, an inverse relationship between valve height, *H* and *R*_c_ was formed so that height decreases as diameter increases. This reduces the risk of valve protrusion downstream of the aorta and ensures coaptation is retained by stretching longitudinally as it decreases radially. Therefore a *H*/*R*_c_ ratio of 0.71 was used, this is inverse of the commonly reported value of 1.4.^17,18^

**Figure 1.**
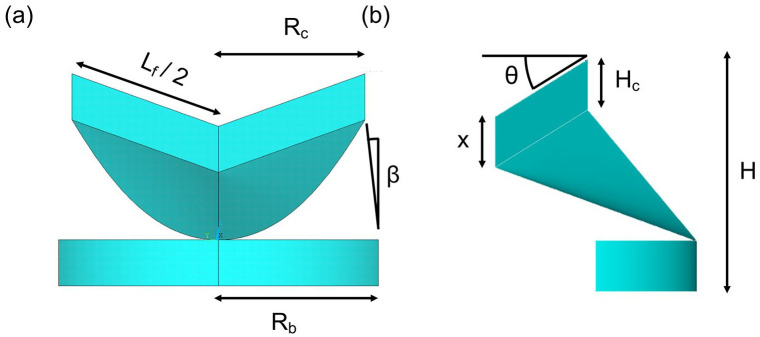
Parametric design of leaflet and stent base, from (a) circumferential and (b) radial perspectives. Valve parameters defined; *l*_f_: leaflet free edge; *R*_b_: radius at base; *R*_c_: radius at commissures; β: angle of stent to the vertical; *x*: coaptation height; θ: angle of free edge to the horizontal; *H*: valve height; *H*_c_: height of the commissural region.

Once all the parameters satisfied the relationship with the valve diameter, the code was imported into Ansys MAPDL 19R2 to generate the surfaces of the coaptation region, leaflet belly and stent base (Figure 1). To determine leaflet deformation, a fluid-structural analysis using an Immersed Boundary (IB) method will be used to cyclically load the valve at decreasing incremental compression from 24 mm to 16 mm. These dimensions correspond to the sinutubular dimensions for a BSA range of 0.95–1.4.^20^

### Meshing and Solution Process

The generated parametric design was imported into an explicit solver (Ansys Explicit Dynamics v19R2) which uses very small time steps to run finite element (FE) simulations of large deformations or high non-linear physics within a short time (<1 s) duration. The geometry was smoothed to prevent highly distorted elements from arising during the solution process. A thin frame with a strut thickness of 0.3 × 0.5 mm was constructed from the line forming the leaflet commissures and leaflet base to form the stent. This stent thickness was selected to maintain a support structure for leaflet anchoring but keep computational costs down. Leaflets were modelled as a 0.5% (w/v) carbon nanotube polyurethane composite using uniaxial test data derived from an earlier study.^15^ To reduce computational costs, the composite leaflet and aorta were modelled having a thickness of 0.1 mm and assumed to have linear isotropic and quasi-incompressible material properties. Full details of all material properties are outlined in Table 1.

**Table 1.** Material properties assigned to the individual geometries.

*Geometry*	Material	Density	Elastic stiffness (MPa)	Poisson’s ratio	Source ref.
*Leaflets*	0.5% MWNT TPU	1150	40.0	0.45	Rozeik et al.^15^
*Stent*	Stainless steel	7850	2.0 × 10^5^	0.3	Ansys lib.
*Arterial wall*	Aorta	1000	0.5	0.49	Kaiser et al.^20^
Blood*	Blood	1060	1.32 × 10^3^	0.49	Kunzelman et al.^21^

* Bulk modulus reduced to 22 MPa to reduce computational costs. This is similar to values used in literature.^21,22^

**Figure 2.**
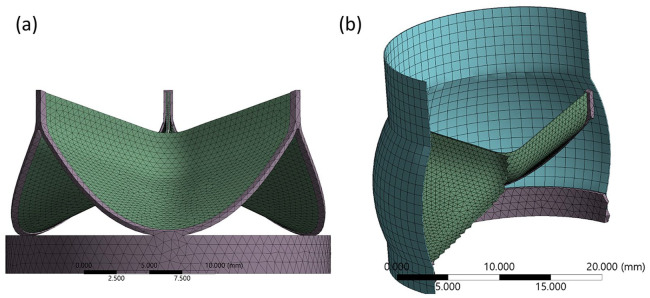
Mesh of structural (Lagrangian) components: (a) perspective view of valve and stent and (b) cross-section of the total mesh generated. Fluid domain omitted for clarity.

**Figure 3.**
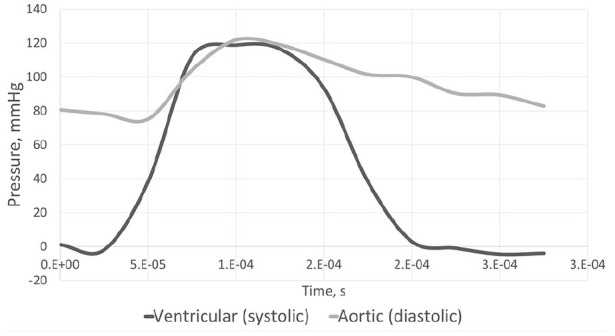
Systolic and diastolic pressure curves derived from Fourier series^23^ and adapted to reflect a cycle time of 2.6 × 10^−4^ s.

In the Immersed Boundary (IB) method, the leaflets, stent and arterial wall are deformable and modelled using the Lagrangian reference field and the blood surrounding the structures was modelled using a Eulerian reference field. Automatic time-stepping was applied with a cardiac cycle time of 2.6 × 10^−4^ s, taken to reduce computational time without compromising results. A small gap of 50 µm was created between the leaflets to prevent bonding prior to the start of the solution process. The leaflets and stent were discretised into tetrahedral elements and the remaining structures were discretised into hexahedral elements, totalling 14,765 elements and 15,920 nodes (Figure 2). A finer mesh was generated for the 18 mm and 16 mm stents to overcome their convergence issues. The outer stent wall is incrementally compressed using a displacement load in the radial direction whilst constraining movement in the axial direction. Leaflets are bonded at the commissures and belly region to the inner surface of the stent. For the diastolic phase, a frictionless contact between leaflet surfaces is applied, but during the systolic phase contact is disabled to avoid convergence issues. As data is only collected once open, its exclusion does not impact the outcome of the study. The diastolic and systolic pressure curves were applied normal to the aortic and ventricular leaflet surfaces respectively and derived from a Fourier series, with the frequency adapted to reflect the time step applied in the solver (Figure 3).^23^ Results were output for radial deformation at peak diastolic and systolic pressures taken following the third diastolic cycle and seventh systolic cycle to ensure solution stability.

## Results

**Figure 4.**
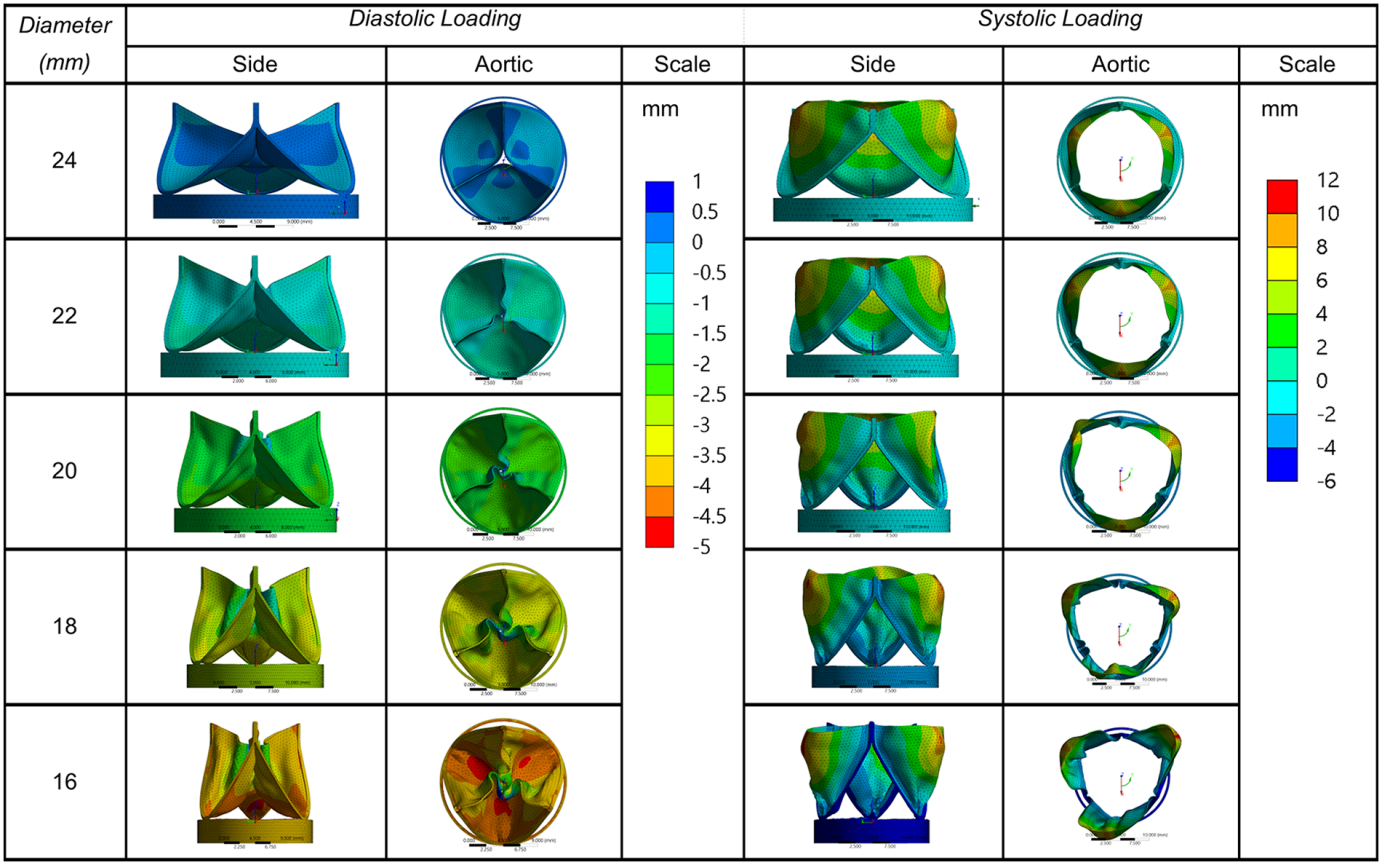
Radial leaflet deformation in mm, from side and top views of valves decreasing from 24 to 16 mm in diameter.

**Figure 5.**
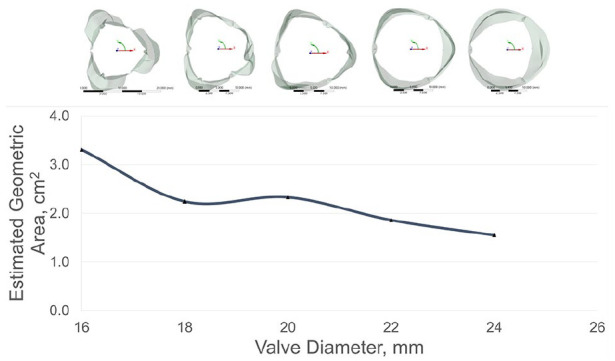
Maximum nodal displacement at the leaflet free edge.

Results were taken of the deformation along the radial direction (Figure 4). As the annulus size decreases, the maximum aperture of the leaflets appears to increase, the height extends along the longitudinal axis and the leaflet belly exhibits some sagging. A degree of leaflet buckling is observed during diastolic loading, which was particularly noticeable when the valve compressed to an 18 mm diameter annulus or less. Maximum radial displacements were taken at three equilaterally distanced nodes on the leaflet free edge to estimate the geometric orifice area (Figure 5).

## Discussion

No paediatric valve to date exists which can expand at a rate mimetic to somatic growth and without necessitating clinical intervention. To our knowledge this is the first in-silico study on the design of a paediatric heart valve, that can expand synchronously with somatic growth as the height varies with annular dimension. Computational modelling is a cost-effective method of identifying early design failure modes, enabling several iterative cycles of design optimisation prior to bench and clinical testing.^24^ Various fluid-structure interaction (FSI) approaches have been studied to numerically model cardiac valves, including the Arbitrary Lagrangian-Eulerian (ALE) approach,^22,23,25^ immersed Boundary (IB) method^26^ and fictitious domain method.^27^ In the ALE-FSI approach, the solid elements (Lagrangian reference field) would be treated as a void and the fluid (Eulerian reference field) adapts around the moving structures using a penalty-based approach. With the IB-FSI approach, the deforming Lagrangian elements overlap the fluid domain. These methods are explained in further detail elsewhere.^28^ Initial attempts to model the ALE-FSI approach with shell elements led to large distortions early in the solve process. As this is a preliminary study, a full ALE-FSI approach would have been computationally expensive to run. Therefore, to keep costs low and avoid convergence issues, a simplified IB-FSI method with solid leaflets was used instead. There are benefits to an FSI approach over FE, as it reduces the distortions in the Lagrangian elements likely to be encountered with large deflections. Future work however will utilise a full ALE-FSI approach to obtain more accurate data on the behaviour of blood parameters including transvalvular pressure gradients, shear stress and paravalvular leakage throughout growth.

Three key issues are discussed in this study; relationship of the leaflet parameters, potential impact on flow dynamics and material properties. Thubrikar et al.^17^ noted that it is desirable for the commissural radius to be slightly smaller than the base radius to provide a wider opening angle. However, a fine balance needs to be struck; a commissural radius that is too small will lead to stenosis, whereas one that is too large can comparably lead to obstruction of blood or high shear stresses from excess leaflet material. Additionally, leaflets that protrude too far into the aortic sinuses could prevent blood recirculation from facilitating closure. In this study a ratio of 1.1 was opted, allowing the angle of the stent posts to protrude slightly into the aortic outflow tract. This facilitated a degree of leaflet opening, which was more clearly observed in the 20 mm and 22 mm annular sizes. Indeed, the degree of leaflet expansion increased as the diameter decreased, improving the estimated geometric orifice area (GOA) which may be a direct result of the longer free edge in relation to diameter. There is a risk, however, that an increased GOA could obstruct the coronary ostia. Given height is designed to decrease with growth, this feature may go some way to mitigating the issue. An in-depth study using patient specific growth data of the paediatric aortic sinus would benefit accurately investigating this phenomenon further.

As the present focus is on leaflet design, the force of the stent on the aortic root was not considered this time. However, stent design is of critical importance, particularly as it needs to adapt to the dynamic changes in our TAV following somatic growth and remain thin enough to achieve a fully percutaneous paediatric TAV. Advances in TAVI technologies have achieved low delivery profiles of 16Fr (14Fr with the Edwards SAPIEN 3 eSheath), improving complications particularly prevalent in smaller arteries such as major vascular bleeding. This will be the central focus of a subsequent study and the impact of stent radial force and restenosis on surrounding tissue structures will be investigated.

As the TAV increases in diameter, the coaptation area decreases which could contribute to paravalvular leakage, this appeared to mildly affect the 24 mm annulus valve. Anisotropic leaflet properties can facilitate coaptation and reduce leakage during diastole. Despite their key role in leaflet dynamics, fibre orientation of native tri-layered leaflets are rarely considered.^27^ Work has previously been reported on the feasibility of fibre reinforced leaflet materials using carbon nanotubes, which can enable thin leaflets to be developed without compromising strength or flexibility.^15^ Further research in this field will be conducted to control alignment of the nanofibres to enhance the coaptation and expansion performance of the TAV.

This preliminary study set out to investigate the relationship between crucial valve parameters and leaflet deformation following cardiac loading as the annulus dimensions varied. More in-depth analysis is needed to investigate the impact this design has on performance particularly with regards to a dynamic aortic root and flow parameters that change with somatic growth. Our early findings have laid the foundation for the design of a TAV that will expand mimetically with somatic growth. Success in the design optimisation step will lay the groundwork for future bench testing and ultimately be fundamental in reducing the need for multiple revision surgeries, thereby improving paediatric quality of life.

## Limitations

To prevent large solver errors and reduce computational costs, some geometrical components were simplified. The computational limitations are documented below:

1. It is well known that dynamic expansion of the aortic annulus occurs during the normal cardiac cycle. However, the radial directional displacement applied to the stent and aortic surfaces limited this. A future study will investigate the effect of dynamic annulus expansion on the leaflet coaptation.
2. As compression of the aortic wall would lead to a thicker wall at smaller diameters, the aorta was given a thickness of 0.1 mm to mitigate this. This could consequently have eliminated the effects of elastic rebound on blood flow, which may have a key role in leaflet coaptation.
3. The leaflet and aorta were assumed to have linear elastic material properties. This may likely impact leaflet coaptation, where radial forces dominate to facilitate closure. Work is currently underway to investigate the effect of non-linear material properties on valve mechanics.

## Conclusion

To our knowledge, no attempt has yet been made to develop a paediatric heart valve that can expand in size without necessitating intervention for dilation. In this study, the following was achieved:

1. An expandable paediatric valve was developed using parametric design that can radially expand as the height decreases, thereby eliminating the need for additional clinical intervention.
2. Full opening of the leaflets, particularly at smaller annulus sizes was achieved due to the angle of stent posts to the vertical.

Work is ongoing to optimise valve design for the paediatric patient cohort further to minimise leaflet buckling. This will include analysis of the full dynamic aortic root and non-linear anisotropic leaflet structure.
